# Early detection of cerebral sinus venous thrombosis in an extremely low birth weight infant using cranial ultrasound—case report and literature review

**DOI:** 10.1007/s00381-024-06659-3

**Published:** 2024-11-27

**Authors:** Aleksandra Skubisz, Anna Tomaszkiewicz, Małgorzata Bocheńska, Witold Błaż, Luca Antonio Ramenghi

**Affiliations:** 1 Clinical Department of Neonatology and Neonatal Intensive Care Unit, Saint Jadwiga the Queen Clinical Provincial Hospital No. 2, University of Rzeszów, 35-301 Rzeszów, Poland; 2 Department of Neonatology, Poznan University of Medical Sciences, 33 Polna Street, 60-535 Poznan, Poland; 3 Department of Pediatrics, College of Medical Sciences, Institute of Medical Sciences, University of Rzeszów, Rzeszów, Poland; 4 Neonatal Intensive Care Unit, IRCCS Giannina Gaslini Institute, 16147 Genoa, Italy

**Keywords:** Cranial ultrasonography, Brain injury, Premature infant, Extremely low birth weight

## Abstract

**Introduction:**

Cerebral sinus venous thrombosis (CSVT) is a serious condition in premature infants. Early diagnosis is crucial, as untreated CSVT can progress to severe complications such as delayed-onset intraventricular hemorrhage (IVH), which could lead to poor outcomes in this population.

**Research question:**

This case highlights that serial cranial ultrasound can detect CSVT early, enabling prompt treatment and preventing subsequent complications.

**Methods and materials:**

We present the case of an extremely low birth weight infant diagnosed with CSVT based on cranial ultrasonography findings. The patient had no clinical symptoms or previously detected brain injury.

**Results:**

Anticoagulant therapy was initiated immediately after diagnosis. Clot resolution was observed on cranial ultrasound after 5 days and confirmed by magnetic resonance imaging (MRI) on the 82 day of life (36 + 4 weeks of gestational age).

**Conclusion:**

This case shows the significant value of cranial ultrasound as a diagnostic tool in premature infants when MRI is not immediately available. Early detection and treatment using ultrasound may help prevent severe complications.

## Introduction

Cerebral sinus venous (sinovenous) thrombosis (CSVT) is a focal or diffuse disruption of cerebral blood flow resulting from the occlusion of cerebral veins and/or sinuses [1]. According to the literature, the annual incidence of neonatal CSVT ranges from 0.67 to 12 cases per 100,000 live births [2–4]. Estimating the incidence of CSVT in infants is challenging due to its rarity, nonspecific clinical presentation, and differences in the frequency of neuroimaging performed in the first days after birth. Cerebral sinus venous thrombosis is characterized by the obstruction of cerebral venous outflow, which disrupts normal venous drainage and initiates a cascade of pathological processes that lead to increased intracranial pressure and edema. This elevated venous pressure can compromise collateral circulation, potentially resulting in ischemia and venous infarction, which may be accompanied by hemorrhage, especially if thrombosis occurs within the dural sinus system, which includes the transverse sinus [5]. Recent studies have identified several factors that may increase the risk of CSVT. Both maternal and fetal/neonatal risk factors are considered including preeclampsia, placental disorders, mode of delivery, disturbances within Virchow’s triad, coagulation disorders, hypoxia, infections, and dehydration [1, 3, 4]. The majority of CSVT cases are diagnosed in in term infants [3, 6, 7]. Commonly reported clinical symptoms include neonatal encephalopathy, seizures, and more frequently, nonspecific signs such as poor feeding [1, 7]. Diagnosis is particularly challenging in the early stages of thrombosis, when clinical symptoms may be entirely absent [8]. The location and size of CSVT can result in variable clinical presentations depending on the gestational age and underlying cause. In preterm infants, CSVT should be considered in every case of late-onset intraventricular hemorrhage (IVH) or white matter injury (WMI) [9]. Over a quarter of premature infants with CSVT are likely to be asymptomatic [10]. The availability of advanced ultrasound probes and devices, as well as color Doppler systems, has increased diagnostic sensitivity and allowed for better detection of clots/loss of flow in the vessels [11]. During the diagnosis of CSVT associated brain injuries may sometimes be observed. For this reason, some authors still recommend MRI confirmation to achieve a comprehensive diagnosis [1]. The treatment of CSVT in infants remains controversial due to the lack of large randomized trials evaluating the safety and efficacy of therapeutic interventions [12]. Anticoagulation with low molecular weight heparin (LMWH) may be considered in cases without significant intracerebral hemorrhage, progressive thrombosis, or underlying prothrombotic disorders [3, 13]. Evidence on outcomes and prognostic factors in infants with CSVT is limited and inconsistent [1]. The lack of standardized neurodevelopmental follow-up and the high prevalence of associated brain lesions complicate the interpretation of available data. Nevertheless, current literature indicates a mortality rate ranging from 6 to 19%, with neurodevelopmental impairment observed in 40 to 80% of cases [3, 13].

We present the case of an extremely low birth weight premature infant who developed CSVT in the third week after delivery, with no prior visible cerebral pathology or clinical signs.

## Case description

A male infant was born prematurely at 24 + 5 weeks of gestation by cesarean section due to a threatening intrauterine infection, with a birth weight of 780 g. There was no history of maternal coagulation disorders, confirmed chorioamnionitis, or other placental pathologies. The Apgar scores were 3, 5, 7, and 7 at 1, 5, and 10 min of life, respectively. Delayed cord clamping for 1 min was applied. Following delivery, respiratory support was initiated, and the infant was intubated at 3 min of life, receiving the first dose of surfactant. After achieving a stable heart rate and oxygen saturation, the patient was transported to the Neonatal Intensive Care Unit (NICU). Upon admission, the infant’s condition was unstable. The patient was treated with catecholamines and hydrocortisone for hypotension. Arterial and venous umbilical catheters were placed. Lung ultrasound revealed respiratory distress syndrome (RDS), leading to a repeat dose of surfactant several hours later. Echocardiography indicated pulmonary hypertension, and nitric oxide therapy was initiated. No abnormalities were observed on cranial ultrasound or abdominal cavity examination. The patient remained on mechanical ventilation in volume guarantee synchronized intermittent mandatory ventilation (SIMV/GV) mode and completed a full course of acetaminophen therapy for patent ductus arteriosus. On the eighth day of life (DOL), the patient was diagnosed with late-onset sepsis, with bacterial culture identifying *Staphylococcus haemolyticus*. Cerebrospinal fluid (CSF) was collected, and results did not indicate a central nervous system infection [14]. Antibiotics consistent with the antibiogram were administered. The patient underwent cranial ultrasound at 1, 2, 6, 9, and 14 DOLs, none of which detected any pathology.

**Fig. 1:**
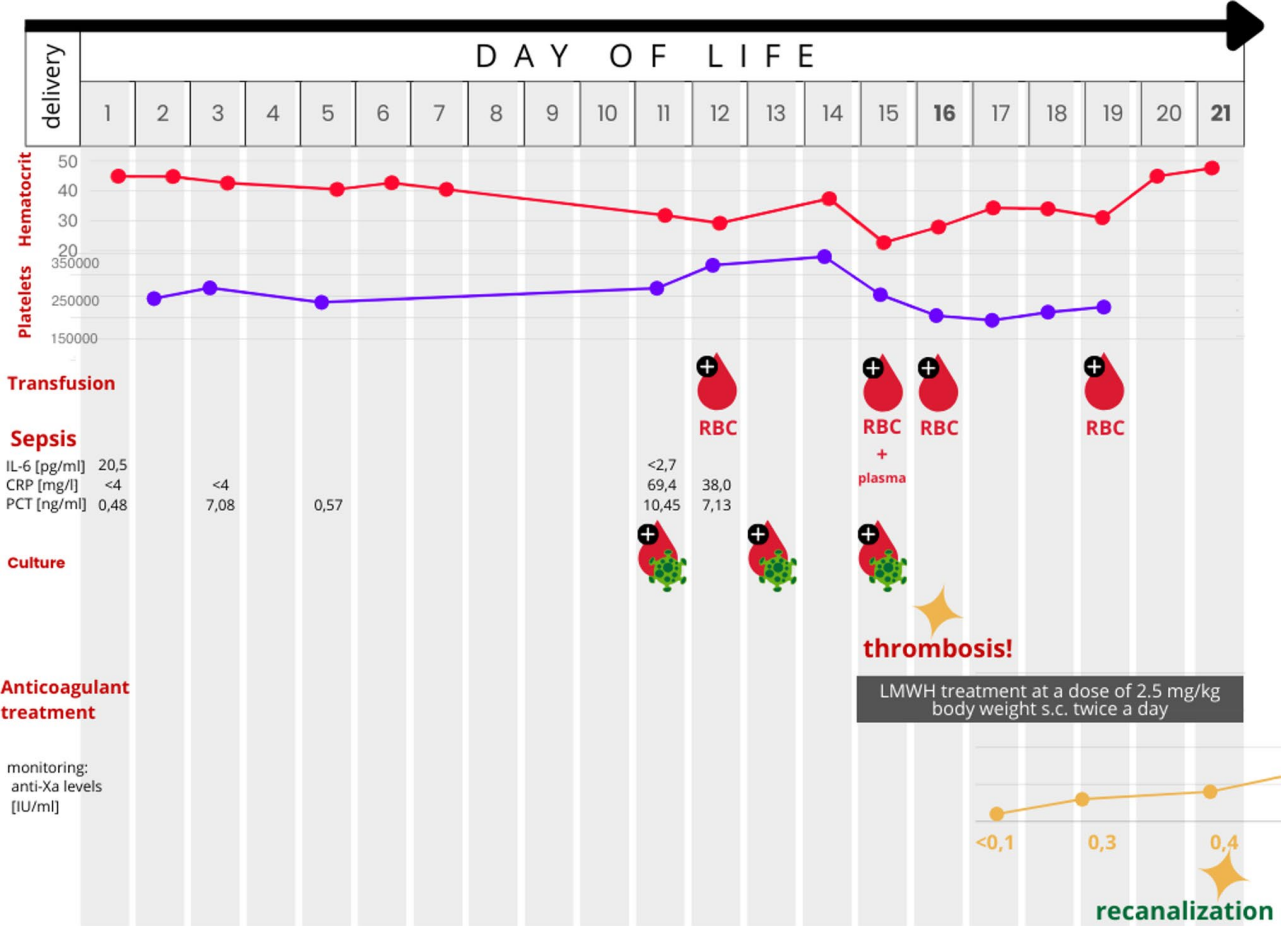
Figure showing the timeline and different test results received on particular days of the patient’s life

On the 15th DOL, during an umbilical catheter dressing change, increased bleeding occurred without other sources or signs of local bleeding. Laboratory tests showed a decrease in hemoglobin (from 11.6 to 8.0 g%) and platelet count (from 345,000 to 204,000/μL). Consequently, red blood cell and plasma transfusions were administered, followed by a second red blood cell transfusion the next day (Fig. 1).

**Fig. 2:**
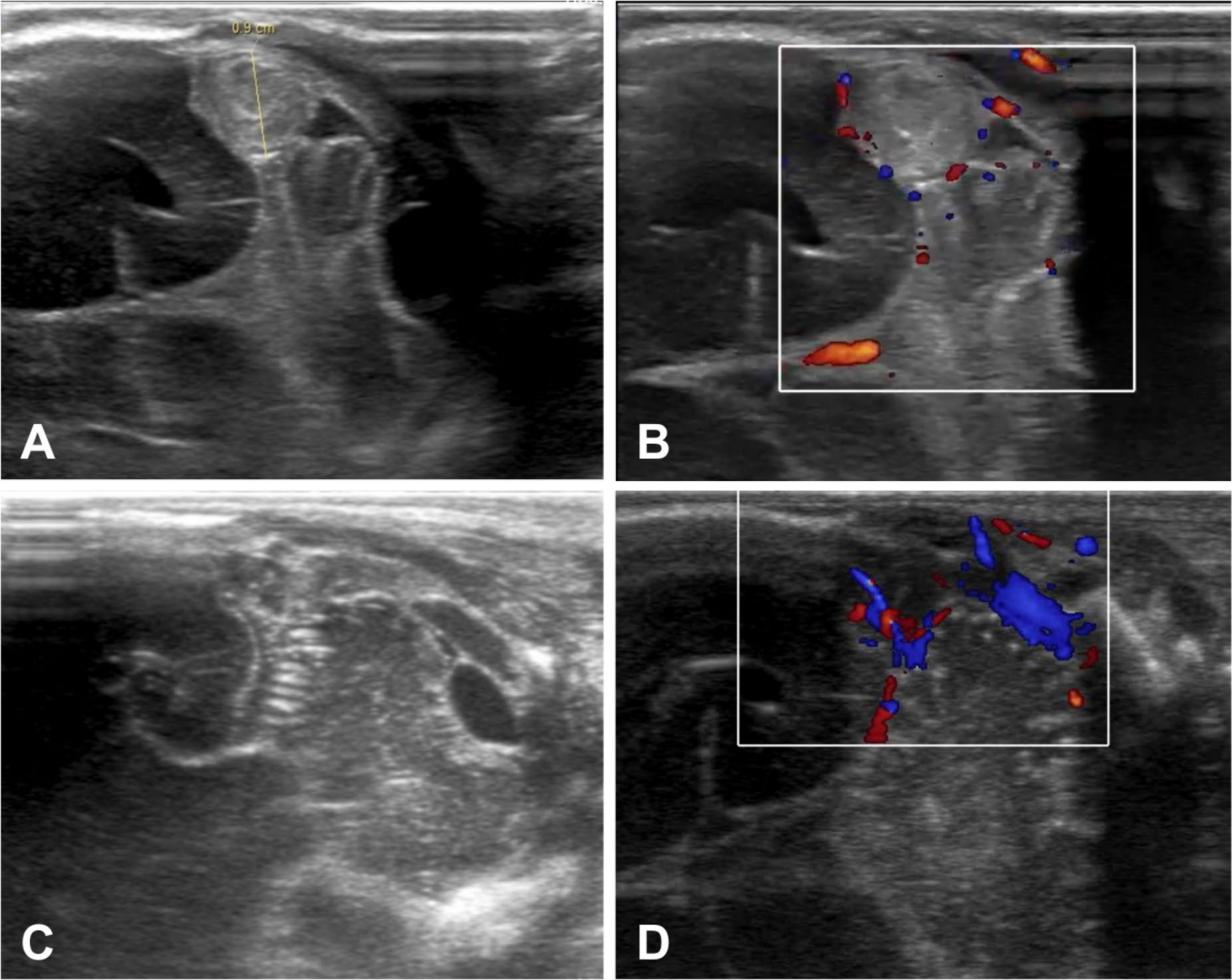
Cranial ultrasound imaging (diagnosing). A, B Ultrasound images taken during exam on the 16th day of life on right side (the day of CSVT diagnosis). C, D Ultrasound images taken during exam on the 16th day of life on left side

**Fig. 3:**
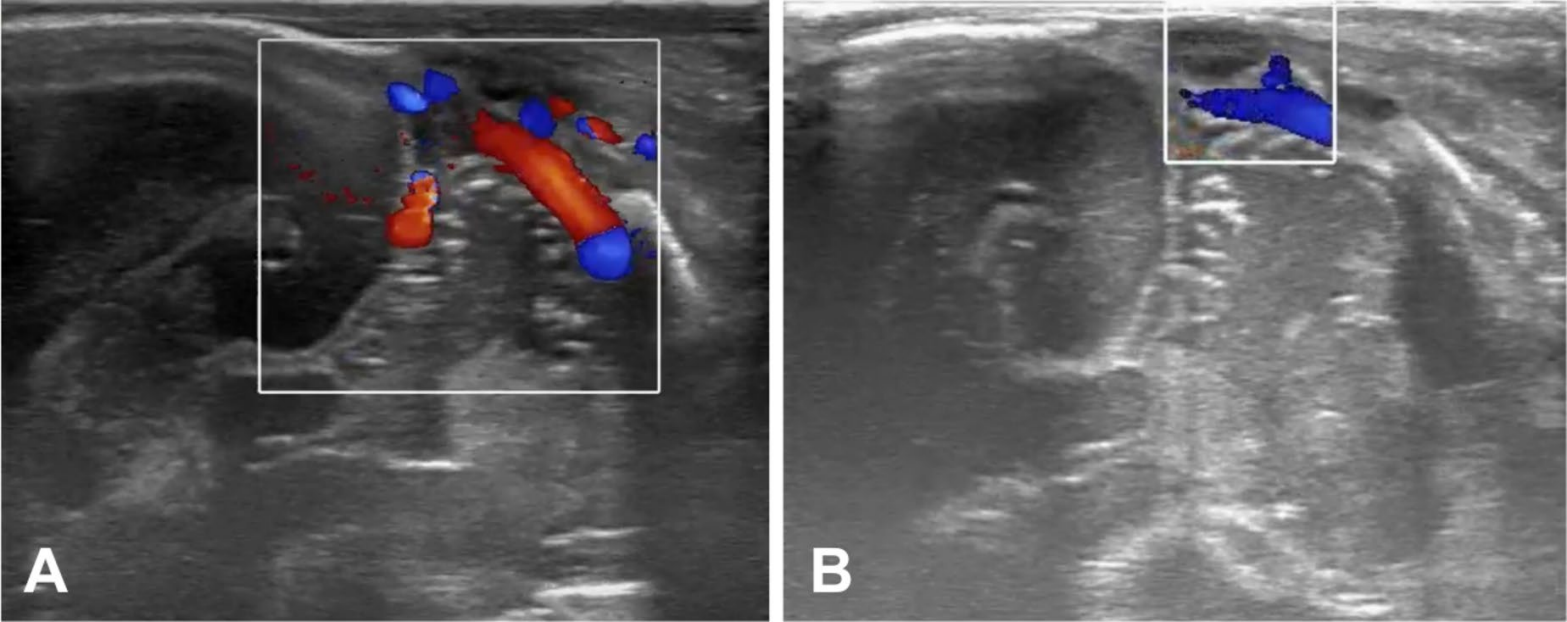
Cranial ultrasound imaging (recanalization). A Ultrasound images obtained during the exam on the 40th day of life (the day of recognition of thrombus resolution) on right. B Ultrasound images obtained during the exam on the 40th day of life (the day of recognition of thrombus resolution) on left side

**Fig. 4:**
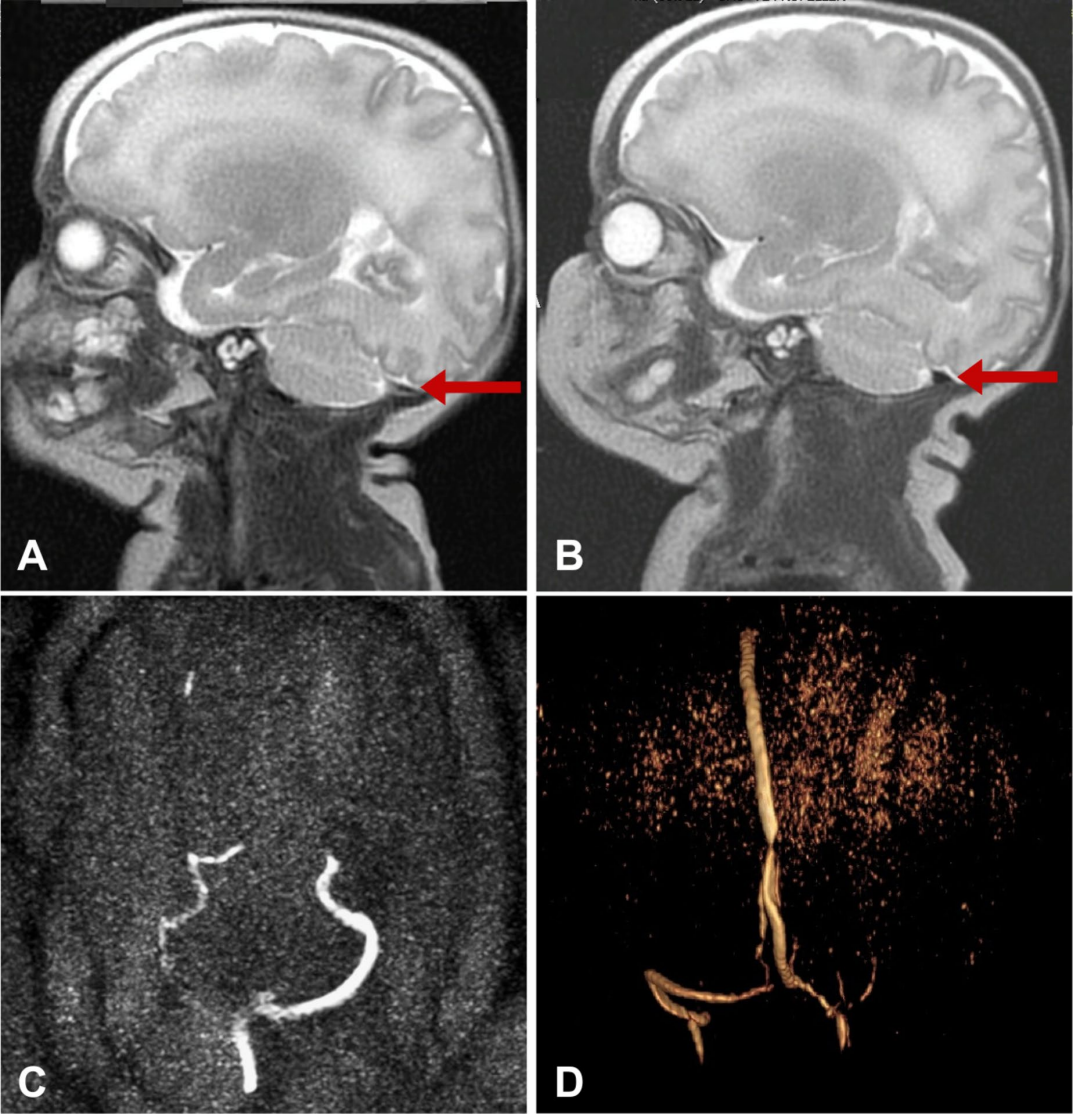
Magnetic resonance imaging. A MRI scan, performed on the 82th day of life, right sagittal cross-section, red arrow points to transverse sinus. B MRI scan, performed on the 82th day of life, left sagittal cross-section, red arrow points to transverse sinus. C, D MR venography with visible flow in both transverse sinuses, markedly smaller on the right side

On the 16th DOL, a control cranial ultrasound of the posterior fossa revealed ongoing transverse sinus thrombosis, predominantly on the right side, with no additional brain injury (Fig. 2). MRI could not be performed that day. The decision was made to start LMWH treatment at a dose of 2.5 mg/kg body weight subcutaneously twice daily, with anti-Xa level monitoring (Fig. 1). Daily cranial ultrasounds were used to monitor the brain and sinuses during anticoagulation therapy. On the 21st DOL, Doppler imaging visualized flow and recanalization of the right transverse sinus (Fig. 3). On the 82nd DOL, magnetic resonance angiography (MRA) revealed a weak but detectable flow signal in the right transverse sinus and right sigmoid sinus, confirming thrombus recanalization. Angio-MR images of the major intracranial arteries were normal, with no visualized vascular malformations or stenosis (Fig. 4).

At a corrected age of 6 months, the patient was assessed using the Hammersmith Infant Neurological Examination (HINE), achieving a score of 75 out of a possible 78, indicating an optimal outcome with no risk of delayed cognitive performance [15].

## Discussion

The presented patient did not present any evident risk factors for CSVT, based on gestational age at delivery and maternal history. Frequent cranial ultrasound monitoring allowed for an early diagnosis and prompt initiation of therapy. This proactive approach resulted in the resolution of the thrombus within 5 after diagnosis. At the follow-up conducted at 6 months of corrected age, neurological examination and HINE scoring showed no signs of psychomotor developmental disorders.

Preterm infants are typically admitted to neonatal intensive care units (NICUs), where they undergo various procedures. The NICU environment, characterized by various stressors, can have adverse effects on these vulnerable infants. This patient was diagnosed with CSVT on the 16th DOL—a timeframe considered a late presentation [17]. The only potential risk factors for CSVT identified in the literature that may have contributed to the development of this condition in our patient’s medical history are hypoxia, hypotension, and a recent episode of sepsis [4, 16, 17].

One of the major challenges associated with the prematurity of our patient is the recognizing of CSVT. Published studies suggest that term-born infants may show a wide spectrum of symptoms [17]. However, the presentation in preterm infants is often nonspecific or may be asymptomatic altogether, as demonstrated in our case [8].

Due to the patient’s prematurity, serial cranial ultrasounds were performed, as recommended by Cizmeci et al., to monitor for the risk of intraventricular hemorrhage (IVH) in this population [18]. This practice enabled the early detection of CSVT in our case, even in the absence of clinical symptoms. Raets et al. confirmed that cranial ultrasound, including color Doppler imaging with scans obtained through the mastoid fontanelle, can detect CSVT at an early stage [8]. This is possible because cranial ultrasound is a fast, bedside, minimally invasive examination that can be performed repeatedly, making it an ideal method for monitoring the brain in preterm infants requiring intensive care. This monitoring is crucial as brain damage can occur within a few hours after delivery or, as in our case, several days later [11]. Therefore, cranial ultrasound appears to be an excellent method for monitoring CSVT in extremely low birth weight infant during treatment, as well as for excluding brain injury after clot resolution. There are reports indicating that the most common consequence of CSVT may be late-onset IVH, but other injuries also occur [19, 20]. Hence, there are recommendations for the highest risk immature infants with weekly scans until at least 28 days of life and alternate weeks after that [21].

Another difficult question with the preterm patient with CSVT is whether or not to initiate anticoagulant therapy (ACT), as there are limited safety data and no randomized trials in this population [1, 12, 14, 22]. Our patient, born at 25 + 5 weeks of gestation, qualifies as an extremely preterm infant, representing only 8% of preterm infants with CSVT according to Christensen et al. [10]. In their study, the majority of patients (69%) received anticoagulation therapy, although only 28% of very/extremely preterm infants were treated. We decided to initiate treatment for our patient due to the absence of associated hemorrhage, as well as the thrombosis fully occluding the lumen of the right transverse sinus and disrupting flow in the left one. Although some studies suggest withholding ACT in cases of small or non-occlusive thrombi, and Christensen et al. noted that 85% of patients experience recanalization regardless of ACT, previously published data suggest that without treatment, there is an increased risk of propagation of the thrombus that might lead to associated brain injury with normal outcomes achieved in only 20% of cases [23]. In contrast, anticoagulant treatment can lead to full recanalization in nearly 89% of infants by 3 months [13]. Consequently, we chose to treat our patient primarily to prevent further injury.

## Conclusion

Cranial ultrasound was crucial not only for the early detection of cerebral sinus venous thrombosis, enabling prompt intervention, but also for ongoing monitoring throughout the course of treatment. The administration of anticoagulant therapy resulted in successful thrombus recanalization. Despite the unavailability of immediate MRI, cranial ultrasound provided a safe and effective method for both diagnosis and follow-up. This case highlights the importance of early imaging and timely treatment in improving outcomes in extremely premature infants with CSVT.

## Data Availability

No datasets were generated or analysed during the current study.
